# Is Abdominal Obesity a Risk Factor for the Incidence of Vitamin D Insufficiency and Deficiency in Older Adults? Evidence from the ELSA Study

**DOI:** 10.3390/nu14194164

**Published:** 2022-10-07

**Authors:** Thaís Barros Pereira da Silva, Mariane Marques Luiz, Maicon Luís Bicigo Delinocente, Andrew Steptoe, Cesar de Oliveira, Tiago da Silva Alexandre

**Affiliations:** 1 Department of Gerontology, Federal University of São Carlos, Rodovia Washington Luís, Km 235, SP-310, São Carlos 13565-905, São Paulo, Brazil; 2 Postgraduate Program in Physical Therapy, Federal University of São Carlos, Rodovia Washington Luís, Km 235, SP-310, São Carlos 13565-905, São Paulo, Brazil; 3 Postgraduate Program in Gerontology, Federal University of São Carlos, Rodovia Washington Luís, Km 235, SP-310, São Carlos 13565-905, São Paulo, Brazil; 4 Department of Epidemiology and Public Health, University College London, Gower Street, London WC1E 6BT, UK

**Keywords:** 25(OH)D, abdominal obesity, aging, incidence, vitamin D, waist circumference

## Abstract

Cross-sectional studies have demonstrated an association between abdominal obesity, determined by waist circumference (WC), and vitamin D (25(OH)D) deficiency in older adults. However, longitudinal evidence is based only on general obesity determined using body mass index (BMI). We investigated whether abdominal obesity is associated with the incidence of 25(OH)D insufficiency (>30 and ≤50 nmol/L) and deficiency (≤30 nmol/L), and whether vitamin D supplementation modifies these associations. We included 2459 participants aged ≥50 years from the English Longitudinal Study of Ageing (ELSA) with 25(OH)D sufficiency (>50 nmol/L) at baseline. Abdominal obesity was defined as >88 cm for women and >102 cm for men. After 4 years, 25(OH)D concentrations were reassessed. Multinomial logistic regression models controlled by covariates were performed. Abdominal obesity increased the risk of the incidence of 25(OH)D insufficiency (RRR = 1.36; 95% CI: 1.01–1.83) and deficiency (RRR = 1.64; 95% CI: 1.05–2.58). These risks were maintained when excluding individuals who took vitamin D supplementation (RRR = 1.38; 95% CI: 1.02–1.88) and (RRR = 1.62; 95% CI: 1.02–2.56). Abdominal obesity is associated with the risk of incidence of low 25(OH)D concentrations. WC seems to be an adequate tool for screening individuals with obesity and at potential risk of developing these conditions.

## 1. Introduction

Aging reduces the cutaneous synthesis of vitamin D, a prohormone that plays an essential role in endocrine-metabolic responses. Vitamin D deficiency affects about 75% of the worldwide older population [1]. Although a consensus to classify serum 25-hydroxyvitamin D (25(OH)D) concentrations has not yet been established, the Institute of Medicine (IOM) defines serum concentrations of 25(OH)D < 30 nmol/L as deficient; 30 to 50 nmol/L as insufficient; and >50 nmol/L as sufficient, since such values are capable of supplying the needs of 97.5% of the North American population [2].

In addition to aging, the black race, lower education, smoking, physical inactivity, periods of low sun incidence (autumn and winter), living alone, and a lower consumption of important sources of vitamin D (salmon, oily fish, cod liver oil, and mushrooms) [3,4,5,6] are associated with serum 25(OH)D deficiency. Moreover, 25(OH)D can become trapped in adipose tissue due to the strong expression of vitamin D receptors in this tissue, leading to a significant reduction in the bioavailability of the prohormone [7,8,9,10]. Thus, obesity, especially when restricted to the abdominal region, which is common during the aging process, is another determinant factor of inadequate serum 25(OH)D concentrations in older adults [3,4,5,11,12].

Cross-sectional studies report associations between higher body mass index (BMI), waist circumference (WC), skin folds, and lower serum 25(OH)D concentrations [7] in 453 individuals aged ≥65 years, as well as an association between abdominal obesity (WC ≥ 90 cm for men and ≥80 cm for women) and serum 25(OH)D deficiency (<50 nmol/L) in individuals aged between 20–70 years [13]. However, the few longitudinal studies on this topic do not consider abdominal obesity and present discrepant results. Ding and collaborators [8] found that an increase in BMI, the percentage of trunk fat, and the waist-to-hip ratio (WHR) were associated with the risk of serum 25(OH)D deficiency (<50 nmol/L) in older people in 2.6 years of follow-up. In contrast, González-Molero and collaborators [14] found no association between obesity (BMI ≥ 30 kg/m^2^) and the risk of serum 25(OH)D deficiency (<50 nmol/L) in individuals aged 18–77 years in a six-year follow-up period.

Given the possibility of coexistence of a normal BMI and visceral fat accumulation [15], WC stands out as a more adequate measure for investigating the association between abdominal obesity and the incidence of low concentrations of 25(OH)D in older adults. However, this analysis has not been explored longitudinally. Moreover, vitamin D supplementation could increase 25(OH)D concentrations in individuals with obesity, leading to an underestimation of such associations. Therefore, we have tested the following hypotheses in the present study: (i) abdominal obesity is associated with the risk of 25(OH)D insufficiency and deficiency later in life, and (ii) vitamin D supplementation modifies the effect of these associations.

## 2. Materials and Methods

### 2.1. Study Population

The English Longitudinal Study of Ageing (ELSA) is a panel study conducted in England with a representative sample of community-dwelling individuals aged ≥50 years [16]. The study commenced in 2022. The interviews were conducted biannually and the health examination, i.e., nurse visit, occurs every four years for the collection of blood samples and anthropometric data, as well as the application of performance tests.

**Figure 1:**
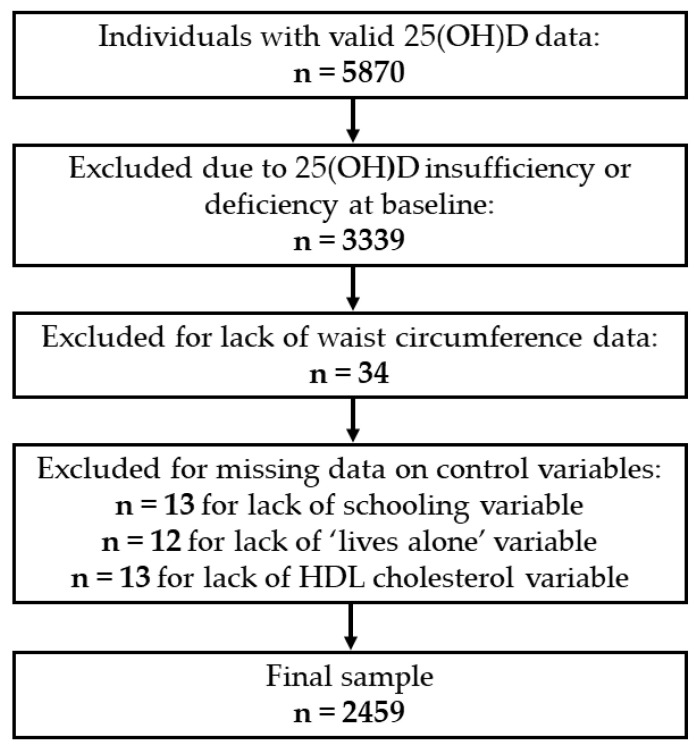
Selection of individuals at baseline (2012–2013). 25(OH)D, 25-hydroxyvitamin D.

The baseline for the present study was wave 6 of the ELSA Study (2012–2013), as it was from this period that serum 25(OH)D concentrations began to be collected. Wave 6 was composed of 9169 individuals, 5870 of whom had valid 25(OH)D data. A total of 3339 individuals were excluded due to serum 25(OH)D insufficiency or deficiency at baseline. Thirty-four others were excluded due to a lack of information on abdominal obesity, and 38 were excluded due to a lack of information on covariates. Therefore, the final analytical sample of the present investigation was 2459 individuals. The sample selection process is shown in Figure 1.

### 2.2. Vitamin D Assessment

Vitamin D was assessed based on serum 25(OH)D concentrations collected by a nurse during the four-year visits. The samples were analyzed at the Royal Victoria Infirmary (United Kingdom). The analyses were performed in duplicate using the DiaSorin Liaison immunoassay, which has an analytical sensitivity of 7.5 nmol/L with a variation coefficient of 8.7% to 9.4%. The lab that performed the analyses is part of the Vitamin D External Quality Assessment Schemes (DEQAS). Serum 25(OH)D concentrations were stratified according to IOM cutoff points: sufficiency (>50 nmol/L), insufficiency (>30 to ≤50 nmol/L), and deficiency (≤30 nmol/L) [2].

### 2.3. Abdominal Obesity

Abdominal obesity was evaluated based on WC measured at baseline using a non-elastic metric tape positioned at the midpoint between the last rib and upper margin of the iliac crest. The measurement was taken twice at the end of the expiration phase of the respiratory cycle. If the measurements differed by more than 3 cm, a third was taken. The mean of the two valid measurements or the two closest if three were taken was used for the analysis. Abdominal obesity was determined as WC > 88 cm for women and >102 cm for men [17].

### 2.4. Covariates

In line with previous studies involving the factors associated with serum 25(OH)D deficiency and obesity [3,4,8], the following covariates were collected at the baseline and incorporated into the study.

The sociodemographic characteristics were gender, age (50–59, 60–69, 70–79, and ≥80 years), race (white or non-white), total household wealth (including financial, housing and physical wealth, such as jewelry and artwork, which were categorized in quintiles), living alone (yes or no) and schooling years, based on the English three-way education system (>13 years; 12–13 years; ≤11 years).

The lifestyle habits considered were smoking status (non-smokers, ex-smokers, or smokers), frequency of alcohol intake (never/rarely, often, daily, or did not answer), and practice of physical activity. Physical activity was assessed using a validated instrument used in the Health Survey for England (HSE), which is based on frequency (“more than once a week”, “once a week”, “one to three times a month” or “almost never”) and intensity of the activity performed (vigorous, moderate, or light) [18]. The combination of this information allowed the classification of individuals in terms of lifestyle as active (light, moderate, or vigorous activity at least once a week) or sedentary (no weekly physical activity) [19].

Clinical conditions were recorded based on self-reports of a medical diagnosis of cancer, lung disease, heart disease, stroke, osteoporosis, osteoarthritis, systemic arterial hypertension, and diabetes. The presence of depressive symptoms was considered when the score on the shortened version of The Center for Epidemiologic Studies—Depression Scale was ≥ 4 (CES-D) [20].

BMI was calculated dividing weight in kilograms by height in meters squared (kg/m^2^) and classified as underweight (<18.5 kg/m^2^), normal weight (≥18.5 and <25 kg/m^2^), overweight (≥25 and <30 kg/m^2^) and obesity (≥30 kg/m^2^) [21].

The biochemical measures were triglycerides (high when ≥150 mg/dL) [22], total cholesterol (high when ≥200 mg/dL), LDL cholesterol (high when ≥100 mg/dL) and HDL cholesterol (low when <40 mg/dL for men and <50 mg/dL for women) [23]. As abdominal obesity is associated with an increase in systemic inflammation, C-reactive protein was considered high when ≥3 mg/L [22].

The season of the year in which the blood samples were collected was another control variable considered and was categorized as spring (March to May), summer (June to August), autumn (September to November), or winter (December to February). The use of vitamin D supplementation and carbamazepine was also considered, the latter of which is an antiseizure agent with the potential to reduce serum 25(OH)D levels [24].

### 2.5. Statistical Analyses

Descriptive statistics were used for the characteristics of the individuals at baseline according to the absence/presence of abdominal obesity. Continuous quantitative variables were expressed as mean and standard deviation values, and qualitative variables were expressed as proportions. Comparisons between individuals with and without abdominal obesity were performed using the chi-square test and Student’s *t*-test. A *p*-value < 0.05 was considered indicative of statistical significance.

To analyze whether abdominal obesity was associated with the risk of incidence of serum 25(OH)D insufficiency and deficiency, multinomial logistic regression models were performed and controlled by sociodemographic, behavioral, clinical, and biochemical characteristics. For such, univariate analyses were first performed with the control variables using the stepwise forward method, and variables with a *p*-value ≤ 0.20 were incorporated into the multivariate models. Those with a *p*-value < 0.05 after the adjustments remained in the final model and were considered significantly associated with the outcome [25].

As individuals who received vitamin D supplementation could be a source of confusion, a sensitivity model was performed excluding such individuals. In all analyses, “individuals without abdominal obesity” and “vitamin D sufficiency” were the reference categories for the comparisons. All analyses were conducted in Stata 16^®^ program (StataCorp, College Station, TX, USA).

## 3. Results

Among 2459 individuals with serum 25(OH)D sufficiency at baseline, the mean age was 66 years (SD ± 8.5), and the majority were women (53.9%), white, had low schooling, were ex-smokers, had frequent alcohol intake, and were physically active. The most prevalent health conditions were osteoarthritis, hypertension, and heart disease. High frequencies of hypercholesterolemia and high serum levels of LDL cholesterol were also found. A total of 10.3% had a medical diagnosis of osteoporosis and only 4.5% received vitamin D supplementation at baseline (Table 1 and Table 2).

The individuals with abdominal obesity at baseline represented 43.6% of the sample and were older, predominantly women, had lower schooling, had lower income, and were more likely to report rarely consuming alcohol compared to those without abdominal obesity. Individuals with abdominal obesity had higher frequencies of hypertension, osteoarthritis, diabetes, high C-reactive protein, low HDL cholesterol, and hypertriglyceridemia, and lower frequencies of high total cholesterol and LDL cholesterol than those without abdominal obesity (*p* < 0.05). In addition, according to BMI, the individuals with abdominal obesity presented a higher prevalence of obesity (Table 1 and Table 2).

In the fully adjusted multinomial logistic regression model, abdominal obesity increased the risk of serum 25(OH)D insufficiency by 36% (RRR = 1.36; 95% CI: 1.01 to 1.83, *p* = 0.043) and the risk of serum 25(OH)D deficiency by 64% (RRR = 1.64; 95% CI: 1.05 to 2.58, *p* = 0.031) (Table 3).

Even with the exclusion of individuals who received vitamin D supplementation in the sensitivity analyses, the results were confirmed, where abdominal obesity remained a risk factor for serum 25(OH)D insufficiency (RRR = 1.38; 95% CI: 1.02 to 1.88, *p* = 0.037) and deficiency (RRR = 1.62; 95% CI: 1.02 to 2.56, *p* = 0.040) (Table 4).

## 4. Discussion

The findings from this large representative sample of English older adults showed that abdominal obesity was associated with the incidence of serum 25(OH)D insufficiency and deficiency after a four-year follow-up period. Moreover, considering those who did not use vitamin D supplementation, the risk of 25(OH)D insufficiency and deficiency among individuals with abdominal obesity was maintained.

According to previous cross-sectional studies [3,4,7,8,12,14,26,27], the higher adiposity demonstrated by both WC and BMI is associated with lower serum 25(OH)D concentrations in older adults. Analyzing 5356 older Irish adults, Laird and collaborators [3] found that obesity (BMI ≥ 30 kg/m^2^) was associated with serum 25(OH)D concentrations < 50 nmol/L. Snjider and collaborators [7] found inverse associations between WC and BMI with serum 25(OH)D concentrations in a sample composed of 453 older adults in The Netherlands. Analyzing a sample of Chinese individuals between 20–70 years of age, Zhang and collaborators [13] found that abdominal obesity (WC ≥ 90 cm for men and ≥80 cm for women) was associated with a greater likelihood of 25(OH)D deficiency (<50 nmol/L) in men and premenopausal women.

In contrast, longitudinal studies offer conflicting results. Ding and collaborators [8] found that increases in the BMI, percentage of trunk fat, and WHR were associated with a greater risk of serum 25(OH)D deficiency (<50 nmol/L) in 859 older adults in Tasmania over a 2.6-year follow-up period. In a study conducted in Spain, however, González-Molero and collaborators [14] found no association between obesity (BMI ≥ 30 kg/m^2^) and a greater risk of serum 25(OH)D deficiency (<50 nmol/L) in 1226 individuals between 18–77 years of age over a six-year follow-up period.

In addition to presenting conflicting results, the longitudinal studies measured obesity by the BMI and WHR, which do not reflect the distribution and accumulation of fat in older adults as well as WC. BMI is not sensitive to changes in the body fat redistribution pattern throughout the aging process. Although better than BMI, the WHR is not sensitive to differences in the distribution of adipose tissue, as the accumulation of fat in women older than 50 years of age is no longer restricted to the hips and thighs, occurring also in the abdominal region. Therefore, the WC seems to be the preferable measure, as it enables an evaluation of adiposity compatible with the obesity profile in both sexes and is a better predictor of visceral adipose tissue compared to BMI and WHR with the advance in age [28,29,30,31]. Therefore, to the best of our knowledge, the present study is the first to identify that abdominal obesity measured by WC is associated with the risk of the incidence of both serum 25(OH)D insufficiency and deficiency in a four-year follow-up period among individuals aged 50 or older.

The present results are possibly justified by the expression of vitamin D receptors in adipocytes, which makes adipose tissue a kind of vitamin D reservoir that sequesters 25(OH)D circulating in the organism, leading to a reduction in its bioavailability and contributing to a greater risk of the development of serum 25(OH)D insufficiency and deficiency in individuals with obesity [11,12,13,14]. Another possible mechanism is the lower conversion of cholecalciferol into 25(OH)D in the liver of individuals with obesity compared to those without obesity. As obesity is associated with non-alcoholic fatty liver disease, the liver in these individuals may have an impaired capacity to metabolize 25(OH)D, interfering in its distribution to the kidneys, where the conversion of 25(OH)D to the active form of vitamin D (1,25(OH)2D) takes place [14,32].

The present findings also demonstrate stability in the effect of abdominal obesity as a risk factor for the incidence of serum 25(OH)D insufficiency and deficiency following the exclusion of individuals who took vitamin D supplementation. Studies have shown that vitamin D supplementation does not seem to have a significant effect on the treatment of obesity and its complications [33,34], which may support the finding of our sensitivity analysis, where the exclusion of individuals who took vitamin D supplementation does not increase the strength of the association between obesity and the risk of incidence of 25(OH)D insufficiency and deficiency.

The present study has strengths and limitations that need to be acknowledged. The strengths were the use of a large representative sample of community-dwelling English individuals aged 50 years or older. Moreover, a wide range of information was included on sociodemographic, behavioral, and clinical characteristics, which enabled rigorous control in the multinomial logistic regression models. The reasonably long follow-up period is another strong point of this study.

Among the limitations, there is the impossibility of extrapolating the results to institutionalized individuals. The losses during follow-up constitute another limitation, but this is an unavoidable occurrence in longitudinal studies. Finally, despite WC not reflecting visceral adipose tissue as directly as more sophisticated methods, such as X-ray absorptiometry (DEXA), computed tomography (CT), and bioelectrical impedance analysis (BIA) [13,35], it is an easy, low-cost measure and an important predictor of metabolic risk [21,36,37,38], and did not impede us from finding the expected associations.

## 5. Conclusions

In conclusion, abdominal obesity was associated with the risk of incidence of serum 25(OH)D insufficiency and deficiency in English older adults, and the effect of this association appears to be confirmed by excluding individuals who were users of vitamin D supplementation. Our findings highlight the importance of early adoption of obesity screening strategies, to avoid its complications, as well as identify those at potential risk of developing 25(OH)D deficiency, enabling adequate treatment for both conditions. Further investigation is still needed to better elucidate the mechanisms involved in the association between abdominal obesity and vitamin D deficiency. It is also important to know how the trajectories of decline in serum concentrations of 25(OH)D occur in individuals with and without obesity.

## Figures and Tables

**Table 1:** Sociodemographic characteristics of 2459 individuals at baseline, ELSA Study (2012).

	Total (n = 2459)	Without Abdominal Obesity (n = 1386)	With Abdominal Obesity (n = 1073)
Age, years	66.6 ± 8.5	66.1 ± 8.6	67.3 ± 8.3 *
Age group, (%)			
50–59	22.1	24.2	19.4 *
60–69	43.0	43.3	42.6
70–79	27.6	25.4	30.4
≥80 years	7.3	7.1	7.6
Gender, women (%)	53.9	49.4	59.7 *
Race, white (%)	98.9	99.1	98.6
Living alone, (%)	14.3	15.2	13.1
Schooling years, (%)			
>13 years	34.2	40.3	26.3 *
12 to 13 years	28.9	28.0	30.0
≤11 years	36.9	31.7	43.7 *
Total household wealth, (%)			
Highest quintile	27.1	31.3	21.6 *
4th quintile	24.1	23.8	24.6
3rd quintile	21.9	21.1	22.9
2nd quintile	15.4	14.4	16.8
Lowest quintile	9.9	7.9	12.4 *
Not applicable	1.6	1.5	1.7

Variables are expressed as mean, standard deviation, and %. * Statistically significant difference compared to the group without abdominal obesity, *p* < 0.05.

**Table 2:** Lifestyle habits, clinical conditions, and biochemical measures of 2459 individuals at baseline, ELSA Study (2012).

	Total (n = 2459)	Without Abdominal Obesity (n = 1386)	With Abdominal Obesity (n = 1073)
Smoking status, (%)			
Non-smoker	39.4	40.3	38.2
Ex-smoker	52.8	50.9	55.4
Smoker	7.8	8.8	6.4
Alcohol intake, (%)			
Never/rarely	15.1	12.8	18.0 *
Frequently	40.9	39.8	42.5
Daily	38.5	42.1	33.9 *
Not applicable	5.5	5.3	5.6
Physical activity, inactive (%)	3.3	2.8	4.0
Clinical conditions, yes (%)			
Systemic arterial hypertension	35.3	27.0	46.0 *
Diabetes mellitus	8.3	5.1	12.3 *
Cancer	5.7	6.0	5.2
Heart disease	15.5	14.8	16.3
Lung disease	12.6	11.3	14.3
Stroke	2.7	2.0	3.5
Osteoporosis	10.3	10.0	10.5
Osteoarthritis	38.1	31.9	46.0 *
Depressive symptoms	8.4	7.7	9.3
Season, (%)			
Summer	31.8	30.2	33.9
Spring	4.8	5.1	4.5
Autumn	46.0	46.8	44.8
Winter	17.4	17.9	16.8
BMI, (kg/m^2^)	27.2 ± 4.5	24.6 ± 2.8	30.6 ± 4.0
BMI, (%)			
Normal weight (≥18.5 and <25 kg/m^2^)	31.5	52.4	4.6 *
Underweight (<18.5 kg/m^2^)	1.1	1.8	0.1 *
Overweight (≥25 and <30 kg/m^2^)	44.3	43.9	44.9
Obesity (≥30 kg/m^2^)	23.1	1.9	50.4 *
Vitamin D Supplementation, yes (%)	4.5	3.8	5.3
Use of carbamazepine, yes (%)	1.8	2.0	1.7
Biochemical measures, (%)			
High C-reactive protein	23.1	15.7	32.8 *
High total cholesterol	62.3	66.2	57.2 *
Low HDL cholesterol	7.7	4.0	12.5 *
High LDL cholesterol	71.7	75.5	66.7 *
High triglycerides	23.1	15.4	32.9 *

Variables are expressed as mean, standard deviation, and %. * Statistically significant difference compared to the group without abdominal obesity, *p* < 0.05.

**Table 3:** Final adjusted model for incidence of serum 25(OH)D insufficiency and deficiency during 4-year follow-up according to abdominal obesity status, ELSA Study (2012–2016).

	Relative Risk Ratio and 95% CI ^1^	
	25(OH)D Insufficiency (>30 to ≤50 nmol/L)	25(OH)D Deficiency (≤30 nmol/L)
Without abdominal obesity ^2^	1.00	1.00
With abdominal obesity ^3^	1.36 (1.01–1.83)	1.64 (1.05–2.58)

^1^ Adjusted by gender, race, vitamin D supplementation, use of carbamazepine, schooling, osteoporosis, age, seasonality, total cholesterol, depression, smoking, and physical activity. ^2^ Waist circumference ≤ 102 cm for men and ≤88 cm for women. ^3^ Waist circumference > 102 cm for men and >88 cm for women.

**Table 4:** Final adjusted model for incidence of serum 25(OH)D insufficiency and deficiency during 4-year follow-up according to abdominal obesity status in individuals without use of vitamin D supplementation, ELSA Study (2012–2016).

	Relative Risk Ratio and 95% CI ^1^	
	25(OH)D Insufficiency (>30 to ≤50 nmol/L)	25(OH)D Deficiency (≤30 nmol/L)
Without abdominal obesity ^2^	1.00	1.00
With abdominal obesity ^3^	1.38 (1.02–1.88)	1.62 (1.02–2.56)

^1^ Adjusted by gender, race, schooling, osteoporosis, age, seasonality, total cholesterol, depression, smoking, and physical activity. ^2^ Waist circumference ≤ 102 cm for men and ≤88 cm for women. ^3^ Waist circumference > 102 cm for men and >88 cm for women.

## Data Availability

The English Longitudinal Study of Ageing data are available to the scientific community from the UK Data Service for researchers who meet the criteria for access to confidential data, under conditions of the End User License http://ukdataservice.ac.uk/media/455131/cd137-enduserlicence.pdf (accessed on 6 October 2022). The data can be accessed from: https://beta.ukdataservice.ac.uk/datacatalogue/series/series?id=200011#!/access-data (accessed on 6 October 2022). Contact with the UK Data Service regarding access to the English Longitudinal Study of Ageing can be made through the website https://www.ukdataservice.ac.uk/about-us/contact (accessed on 6 October 2022), by phone +44-(0)1206-872143, or by email at help@ukdataservice.ac.uk.
